# Biofunctional analysis of *Vitellogenin* and *Vitellogenin receptor* in citrus red mites, *Panonychus citri* by RNA interference

**DOI:** 10.1038/s41598-017-16331-3

**Published:** 2017-11-23

**Authors:** Muhammad Waqar Ali, Zhen-yu Zhang, Shuang Xia, Hongyu Zhang

**Affiliations:** Key Laboratory of Horticultural Plant Biology (MOE), State Key Laboratory of Agricultural Microbiology, Institute of Urban and Horticultural Entomology, College of Plant Science and Technology, Huazhong Agricultural University, Wuhan, 430070 China

## Abstract

*Panonychus citri* is one of the most damaging pests of horticultural crops. Conventional control of this pest population through pesticides has led to the enhanced pest resistance. Management of *P. citri* population through RNAi, is still largely unknown. In oviparous organisms, fabrication and development of yolk protein play a vital role in the reproduction. *Vitellin* (Vn) is the source of eggs storage that helps in proper functioning of *Vitellogenin* (*Vg*) and *Vitellogenin receptor* (*VgR*). *VgR* is very compulsory protein for the development of *Vg* into oocytes. In the current study, *Vg* (*PcVg*) and *VgR* (*PcVgR*) genes were studied and their expressions at different developmental stages were quantified by RT-qPCR. Females treated with dsRNA of *PcVg* and *PcVgR* genes exhibited reduction in gene expression. Down regulation of target genes significantly effected oviposition and reduced the egg laying capacity up to 48% as compared to control (ds-egfp). Synergistic effect of target gene’s dsRNA was also accessed that reduced the egg laying up to 60.42%. Furthermore, combination of target dsRNA on deutonymph and protonymph also resulted in 67% and 70% reduction in eggs, respectively. Synergistic effect of dsRNA at 1000 ng/ul resulted in longer life span as compared to control treatments. This study suggests to develop a new strategy of *P. citri* population control by reducing its reproduction.

## Introduction

*Panonychus citri* (McGregor) (Acari: Tetranychidae) is a major horticultural pest known as citrus red mite^1,2^. It has high reproduction capacity, short life cycle and damages more than 80 species of ornamental trees and plants mainly including almond, pear, rose, citrus and castor bean^3,4^. Traditionlly, many tactics are used to control the pest population but the use of acaricides is the most prominent. Due to adaptive nature and excessive use of acaricides, its strong resistance against many acaricides has been reported^5^. Currently *P. citri* has become the most dangerous pest of citrus as it has developed more than >23000 and >3500 fold resistance in japan^6^ and Chongqing respectively^7–9^. By keeping in mind the adaptability resistivity and short life cycle, it is need of the time to develop new control strategy by targeting the particular gene. The *Vitellogenin* (*Vg*) is a vital gene which is the precursor of *vitellin* (Vn) and provides essential nutrients, including carbohydrates, lipids, amino acids and vitamins, for proper embryo development^10^. Oviparous insect species relies on two fundamental steps: 1) synthesis of *VG* and 2) deposition^11^. In most insects, *Vg* is triggered by juvenile hormone, while it is driven by ecdysteroids in ticks^12^. For the development of ovary in arthropods, *Vg* is synthesized in the fat body and concealed into the hemolymph, followed and taken up via endocytosis through its receptor known as *vitellogenin receptor* (*VgR*). The *VgR* receptor is attached with clathrin-coated pits on the external surface of growth-competent oocytes^11^. The *VgR* belongs to a superfamily of low-density lipoprotein receptor (LDLR). More deep research revealed that LDLR is further classified into five different domains like 1) epidermal growth factor (EGF), 2) the ligand-binding domain constituting of class A cysteine-rich repeats, 3) trans-membrane region, 4) C-terminal cytoplasmic tail containing an internalization signal and 5) O-linked sugar domain^13,14^. Amdam with his colleagues reported that in the social insect (such as the honey bee) *Vg* is directly involved in the somatic pathway, and the disturbance in this pathway may lead to the decline in somatic cells of honey bees. They also declared that depletion in *Vg* plays the significant role in zinc-ligand, which may cause the pycnosis of haemocytes^15^.

Yolk protein and *Vg* are a key factor in the production of yolk and help in nourishment and development of embryo. Yps, in cyclorraphan flies, are different with *Vg*s those are present in non-cyclorraphan insects^16^. The role of *Vg* biosynthesis have been well defined as it plays very critical role in the reproduction of insects and this pathway could help us to make novel gene-specific acaricide^11,17^. Molecular information and bio-functioning of *Vg* and *VgR* had been revealed in two spotted spider mite, *Tetranychus urticae* ^17^. They also justify the function of Vg gene by inferred amino acid sequences, which confined the von Willebrand factor D domain and the GLCG motif, which were reported to be the common features of Vg sequences in insects and ticks. Northern blot analysis did not detect Vg mRNA in the diapause adult females of *T. urticae* ^17^. However, In *P. citri*, only DNA sequences and transcriptional profiles of *Vg* and *VgR* have been identified^18^, while the bio-function of these two genes on the reproduction of this important pests is still largely unknown.

In the present study, we aimed to find and characterize the proper genes functioning linked with female reproduction in *P. citri*. Selection of target genes solely and in combination of oral dsRNAs lead to female infertility, which may help to establish a pest control technique based on RNAi. It is also demonstrated that RNAi is an alternative method of radioactivity and has opened the new door to deal with the challenges against other agricultural pest.

## Results

### Selection of female specific genes

**Figure 1:**
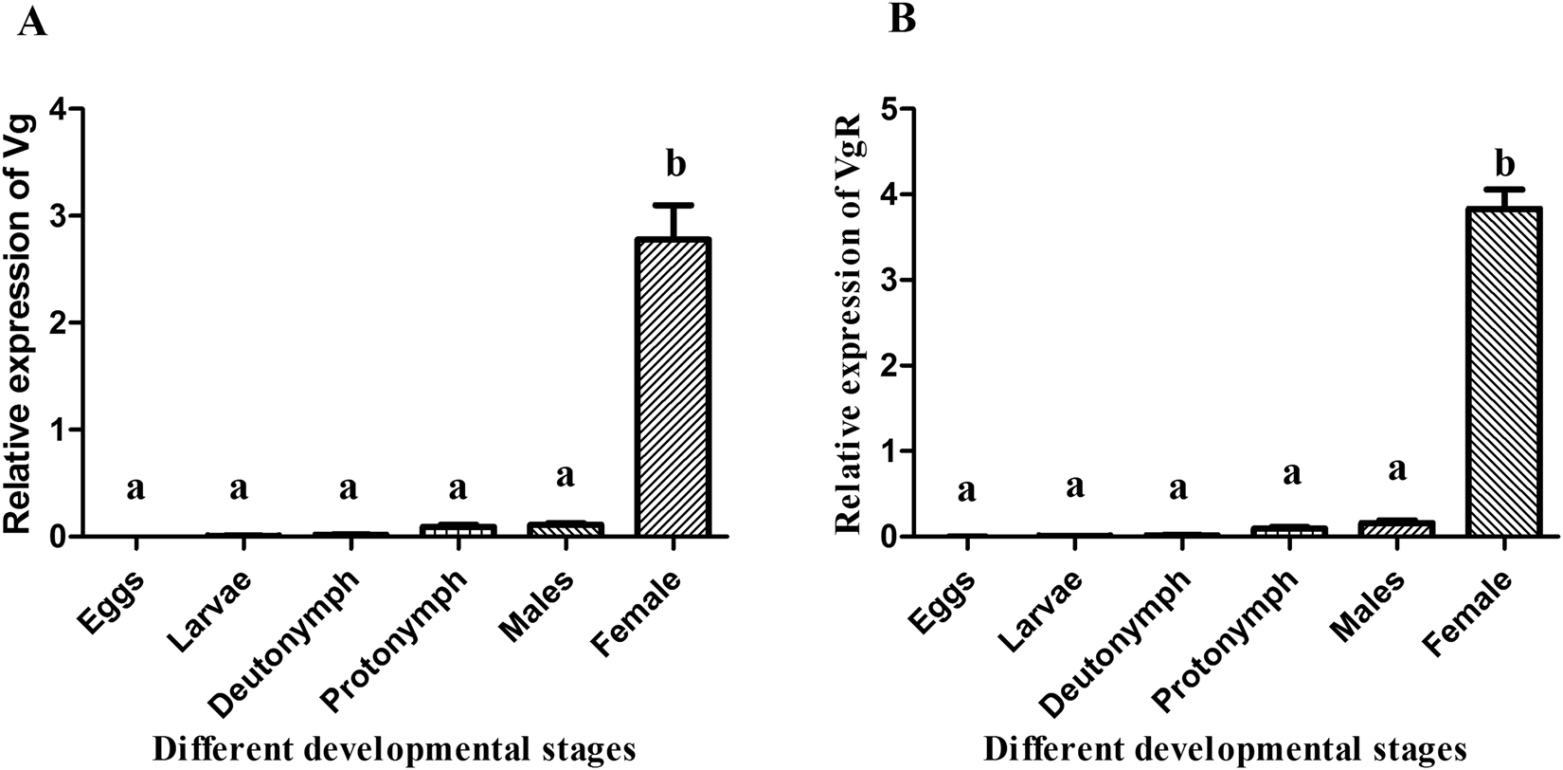
Expression of target genes (*PcVg* & *PcVgR*) in different developmental stages of *Panonychus citri*. Different developmental stages including eggs, larvae, deutonymph, protonymph, male adult, and female adult and different letters indicate significant differences in the expression level at P < 0.0001 according to Tukey’s test following ANOVA analysis.

Target genes were selected based on the previous study^18^. Initially, gene expression was separately assessed in different developmental stages of *P. citri* male and female. Gene expression was analyzed through qRT-PCR by using specific primers (Table S1). Results revealed significantly high expression of target genes (*PcVg* & *PcVgR*) in female adult (Fig. 1). *PcVgR* gene was more dominantly expressed as compared to *PcVg* gene. In male adult, egg, larvae, deutonymph and protonymph, expression of *PcVg* genes was insignificant (0.00009, 0.0021333, 0.0061, 0.032 and 0.060) *PcVgR* (0.0029, 0.0087, 0.0172, 0.0923 and 0.16) (Fig. 1).

### RNAi based gene silencing effects

Target gene in females was confirmed by gene specific primers (Table S2). ORF of target genes *PcVg* and *PcVgR* contain 5553 and 5673 bp nucleotide with molecular weight 210.57 and 211.46 KDa, respectively^18^.

**Figure 2:**
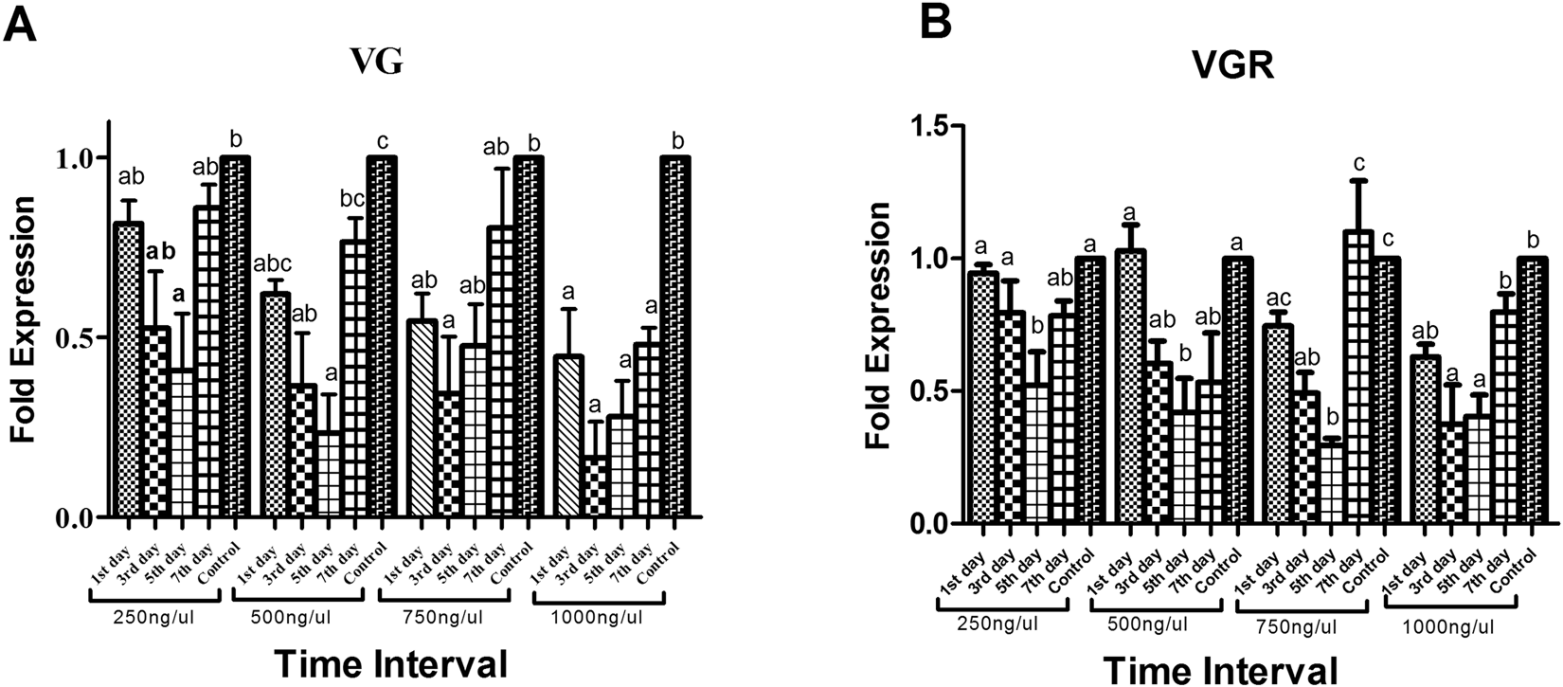
Silencing effect of target genes dsRNAs on different days (1^st^, 3^rd^, 5^th^ & 7^th^) at different concentrations. Feeding by leaf dip method against target genes (*PcVg* & *PcVgR*) (A & B) at different concentrations (250, 500, 750 & 1000 ng/ul) were analyzed. Target gene expression is compared relative to 1 fold of ds-EGFP control. Error bars represent the SE of the mean of three independent biological replicates. All treatments were analyzed as compared to control by using ANOVA Tukey’s-test, P < 0.0001.

qRT-PCR results show that in response to leaf dip method, maximum effect was noted at 1000 ng/ul concentration of both target genes dsRNA. 24-hour post exposure of dsRNA at 1000 ng/ul concentration, *PcVg* & *PcVgR* showed 0.44, 0.63 fold decrease as compared to control ds-Egfp (Fig. 2). While on the 3^rd^ day of treatment target genes showed more down regulation as compared to the 1^st^ day, with 0.27 and 0.37 fold decrease respectively as compared to control. Maximum down regulation of *PcVg* gene was noted at the 5^th^ day of treatment at 500 ng/ul concentration and remained as 0.23 and *PcVgR* showed maximum down regulation against 750 ng/ul concentration of dsRNA 0.29 folds as compared to 1 fold. On the 7^th^ day of treatment, both genes mRNA expression tried to stable its expression and showed the non-significant difference in response to 500 ng/ul. While *PcVgR* gene showed 0.78, 0.53 and 0.29 fold against 250 ng/ul, 500 ng/ul, and 750 ng/ul concentration of dsRNA respectively. In case of 250 ng/ul concentration of both target genes (*PcVg* & *PcVgR*) dsRNA, showed non-significant effect as compared to 1 fold control and was noted as 0.81 & 0.94 respectively (P < 0.0001) (Fig. 2). This non-significant effect is due to very low concentration and very short exposure time.

### Effect on egg laying and hatching rate

**Figure 3:**
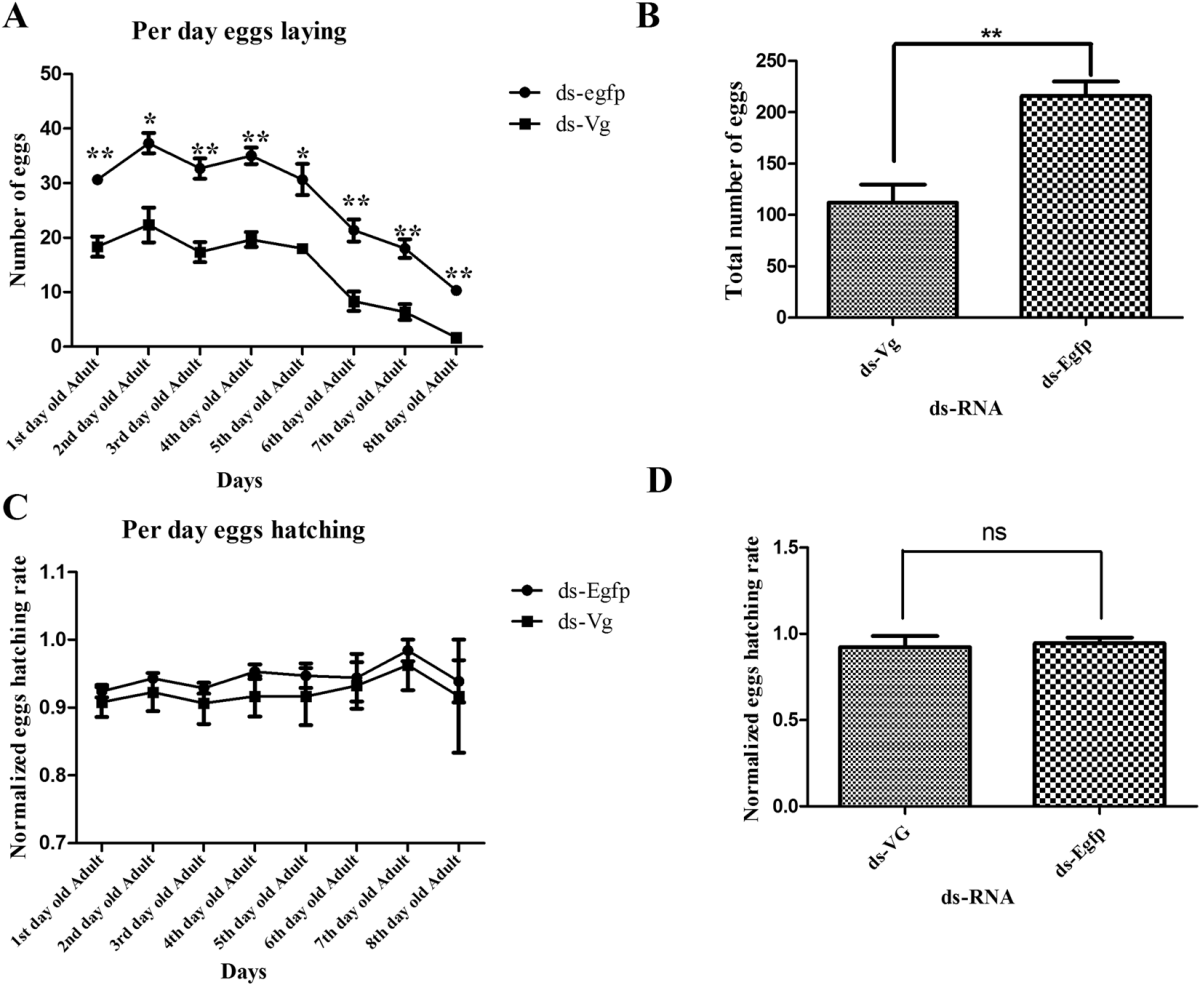
Egg laying and hatching ability in response to 1000 ng/μl of dsRNA. The number of egg laying and hatching capacity of *P. citri* against dsRNA of the target gene (*Vg*) as compared to ds-EGFP.

**Figure 4:**
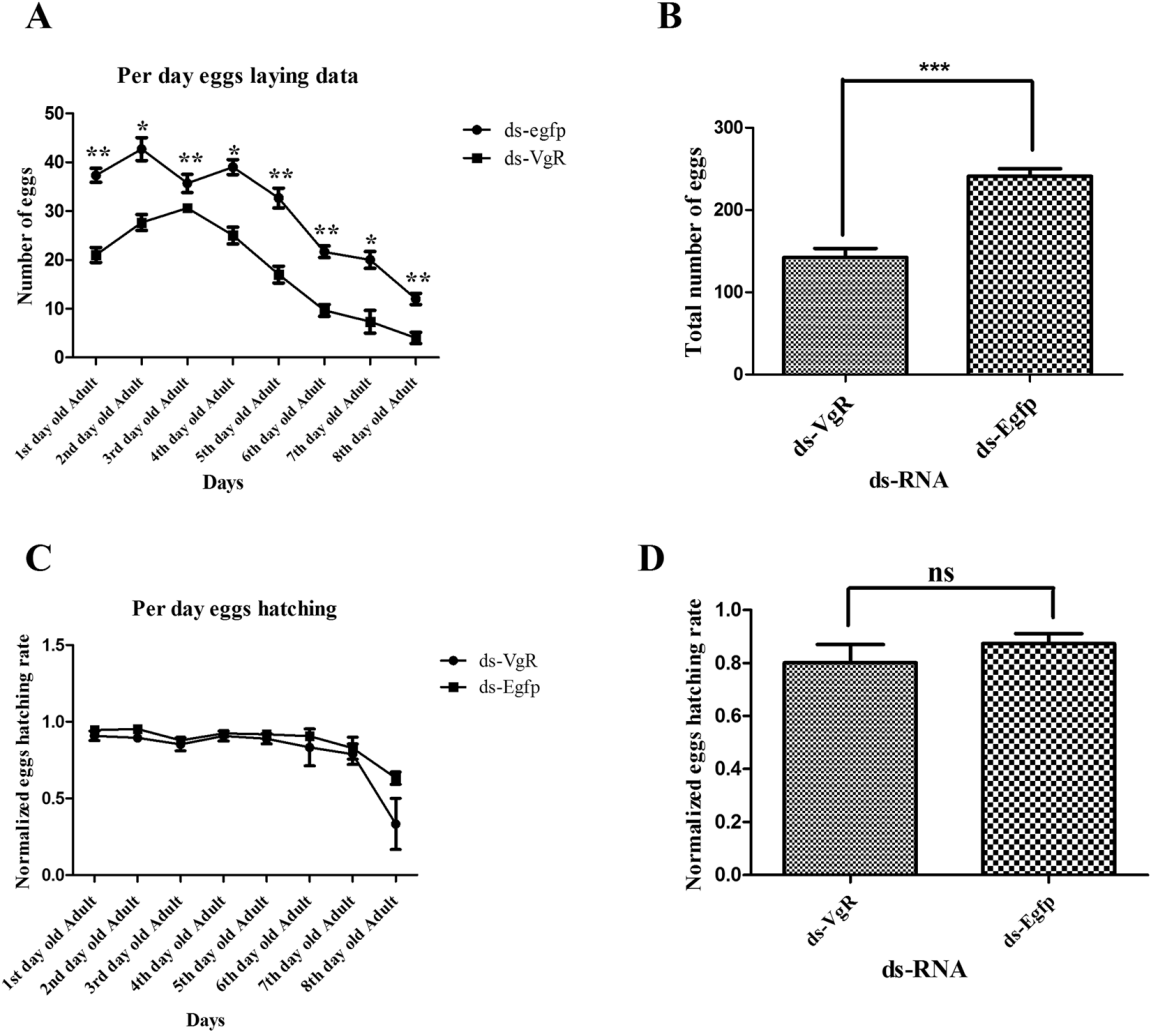
Egg laying and hatching ability in response to 1000 ng/μl of dsRNA. The number of egg laying and hatching capacity of *P. citri* against dsRNA of the target gene (*VgR*) as compared to ds-EGFP.

To verify, whether the silencing of target genes are effective for female infertility, the daily number of egg laying and hatching rate were analyzed against 1000 ng/ul dsRNA concentration of each gene. Egg laying and hatching were accessed from 1^st^-day adult to 8^th^ consecutive days. RNAi of female specific target genes showed high impact on the number of egg laying. Target genes *PcVg* and *PcVgR* dsRNA showed 48.14% and 40.94% female infertility respectively (Figs 3 and 4). In case of egg hatching rate, there no significant difference was noted between treatment and control group (ds-EGFP) (Figs 3 and 4).

### Combined effect of target genes dsRNA on female infertility

**Figure 5:**
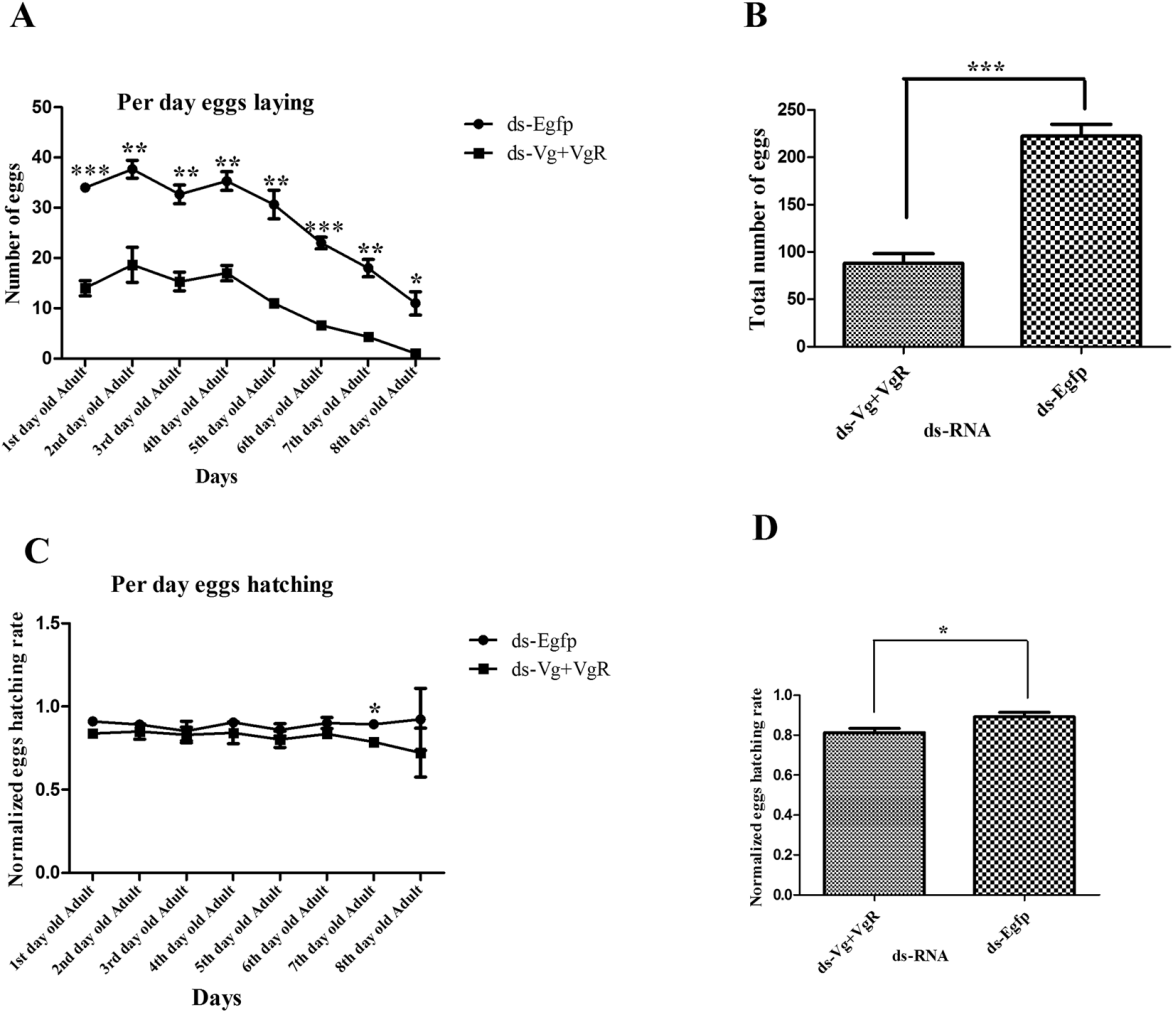
Egg laying and hatching rate in response to combinations of target genes (*PcVg* + *PcVgR*) dsRNA compared with control group (ds-EGFP). Three individual biological replicates were performed, *** indicates the significant difference. One way ANOVA indicate the difference in an average number of laying between all candidate genes dsRNA (P < 0.0001, Tukey test).

To enhance infertility, target genes (*PcVg* & *PcVgR*) dsRNA combination was made at the final concentration of 1000 ng/ul. After synergistic application of dsRNA, the number of eggs significantly reduced as compared to control (ds-Egfp) and individual dsRNA applicatioins. The cumulative reduction in egg laying was noted about 60.42%, against the *PcVg* + *PcVgR* dsRNA as compared to control (Fig. 5).Gene specific product (synergistic effect of target gene dsRNAs) showed the non-significant difference on egg hatching rate but after 7^th^ days of treatment, a slight difference was noted in egg hatching (Fig. 5).

### Technique factors for female infertility based on dsRNA combination

**Figure 6:**
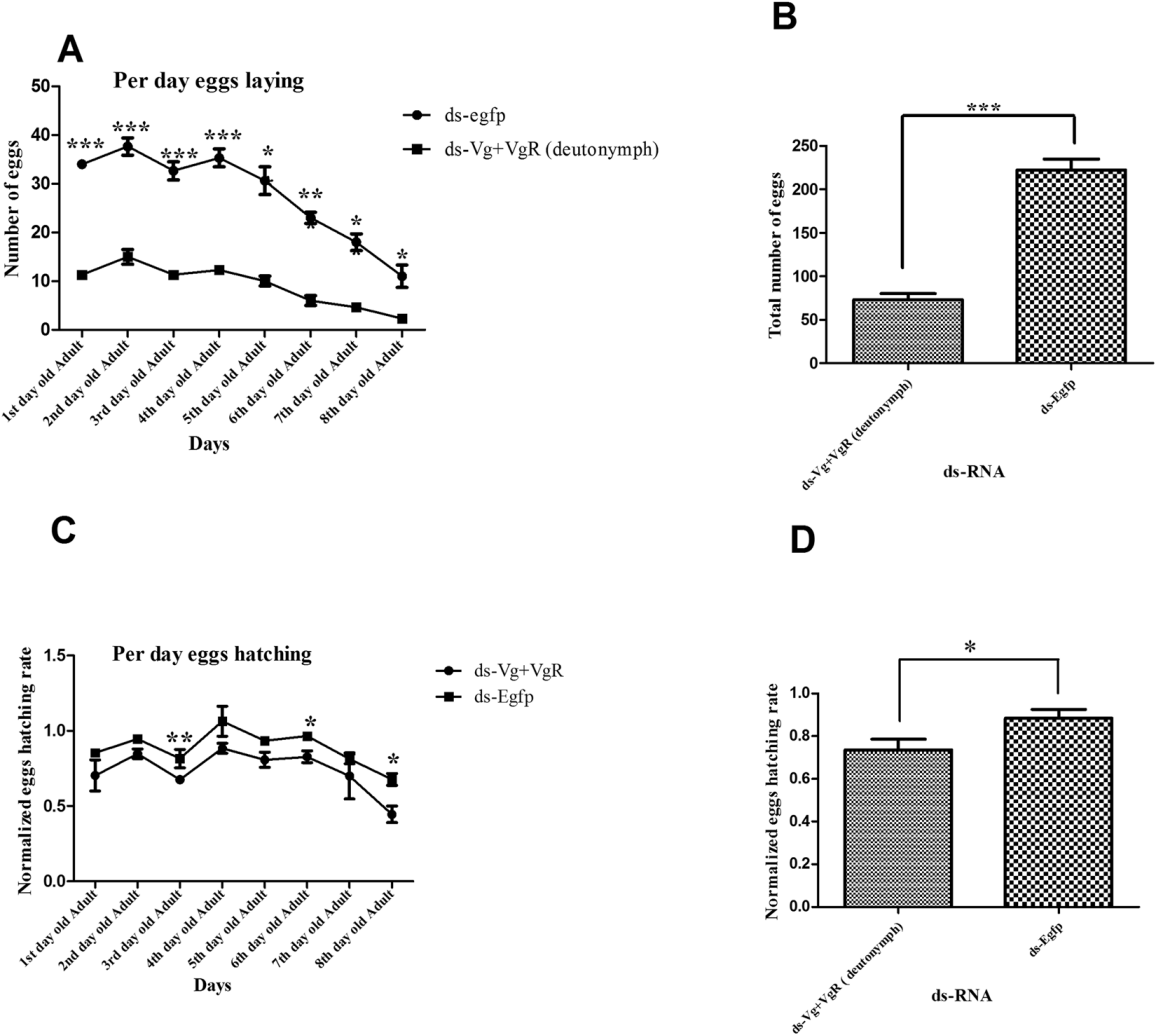
Egg laying and hatching ability in response to 1000 ng/μl of dsRNA (*PcVg* + *PcVgR*) genes in deutonymph of *Panonychus citri*. (**A**) Egg laying/day data between target genes (*Vg* + *VgR*) dsRNA and ds-EGFP. (**B**) Total number of eggs laid by treated mites. (**C**) Egg hatching/day in response of target genes (*Vg* + *VgR*) dsRNA and ds-EGFP. (**D**) Cumulative hatching rate of dsRNA treated mites. One way ANOVA indicates a difference in the accumulative number of egg laying and hatching. Three biological replicates were performed (P < 0.0001, Tukey’s test).

**Figure 7:**
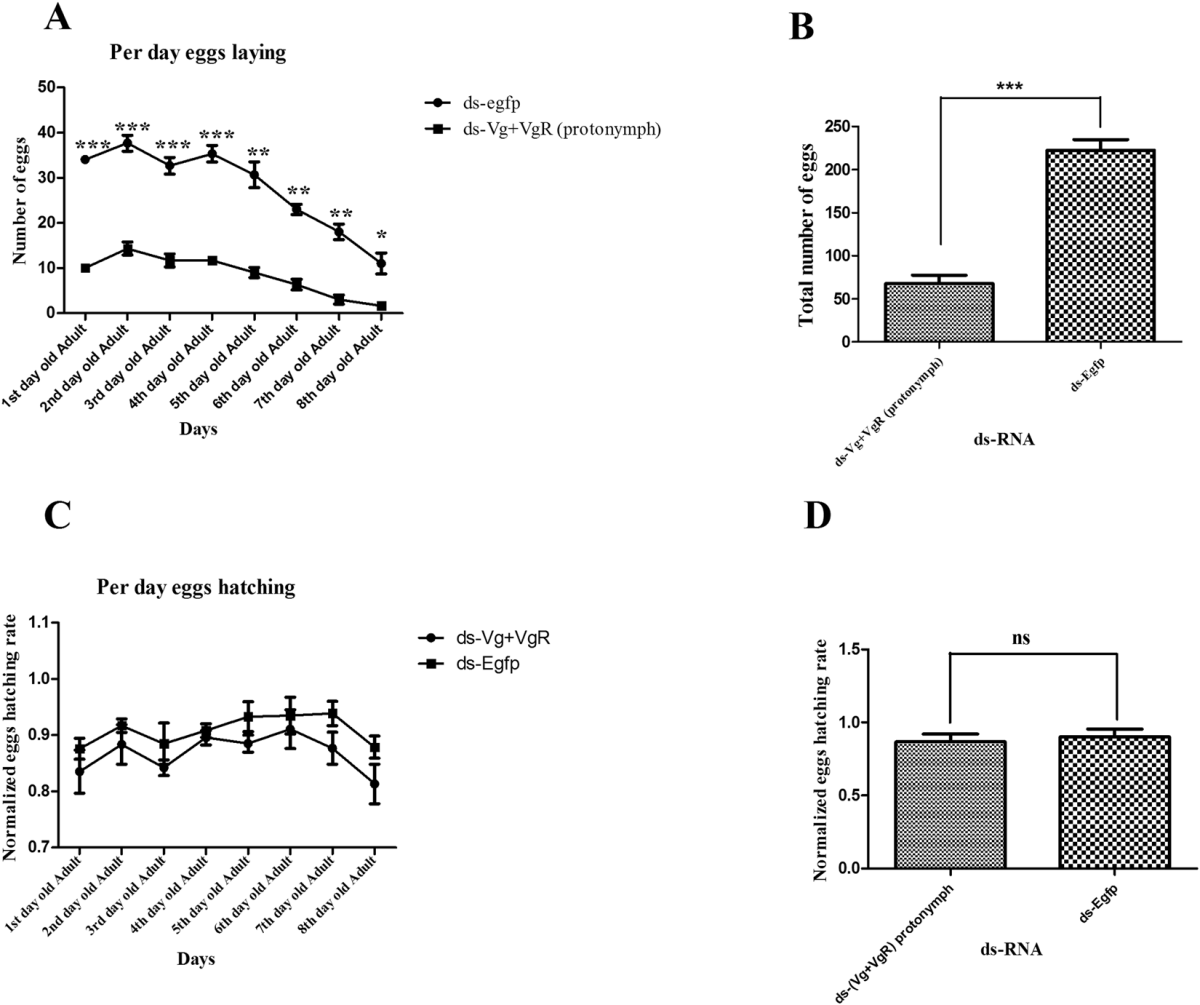
Egg laying and hatching ability in response to 1000 ng/μl of dsRNA (*PcVg* + *PcVgR*) genes in protonymph of *Panonychus citri*. (**A**) egg laying/day between target genes (*PcVg* + *PcVgR*) dsRNA and ds-EGFP. (**B**) Total number of eggs laid by treated mites. (**C**) egg hatching/day in response to target genes (*PcVg* + *PcVgR*) dsRNA and ds-EGFP. (**D**) Cumulative hatching rate of dsRNA treated mites. One way ANOVA indicates a difference in the accumulative number of egg laying and hatching. Three biological replicates were performed (P < 0.0001, Tukey test).

Based on the previous experiments, where synergistic effect of dsRNA caused maximum female infertility, combination of dsRNA was also evaluated against deutonymph and protonymph. In this regard, more than 200 deutonymphs and protonymphs were placed on separate treated leaves and infertility was assessed. Infertility ratio on deutonymph and protonymph was non-significant as compared to each other, but was found more effective as compared to individual dsRNA treatment on adult female and control. As previously described egg laying data of adult mites was noted up to 8^th^ day, cumulative infertility due to deutonymph and protonymph treatment was found to be 70% and 67.2% as compared to control group (ds-Egfp) (Figs 6 and 7). In case of egg hatching, significant difference in treated deutonymph mites was found. Egg hatching was reduced in deutonymph 16.7% as compared to control. Whereas protonymph treated mites showed the non-significant difference in egg hatching (Fig. 7).

**Figure 8:**
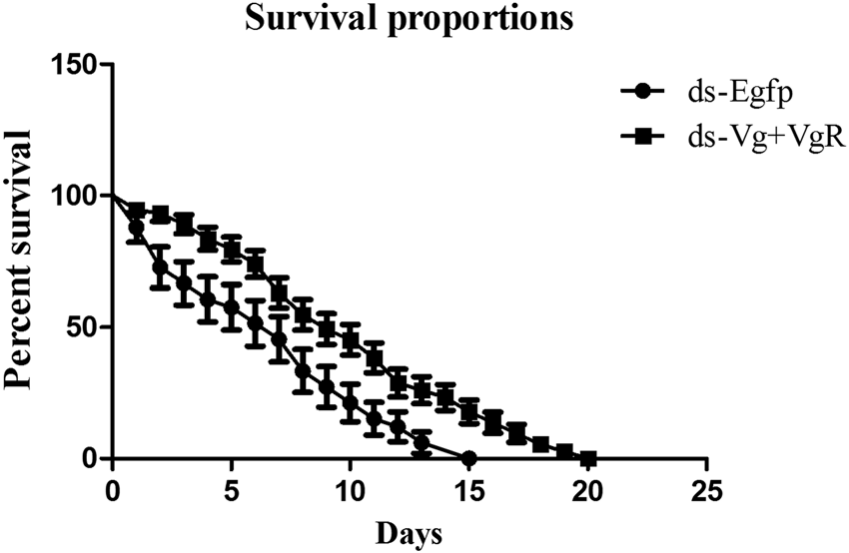
Continuous feeding on treated dsRNA leaves influences the longevity of *Panonychus citri*. Survival curves of *P. citri* after fed with ds-*Vg* + *VgR* and ds-egfp treated leaves. According to Log-rank (Mantel-Cox) Test and Gehan-Breslow-Wilcoxon Test showed highly significant difference *** & ** respectively. The *PcVg* + *PcVgR*-treated mites (n = 200) and the ds-Egfp-control flies (n = 200). Horizontal lines across the scatter diagram represent the mean values.

In the end, the effect of dsRNA combination was evaluated on adult female mites survival rate. Data showed fascinating results that treated mites could survive longer as compared to control mites. According to Log-rank (Mantel-Cox) Test and Gehan-Breslow-Wilcoxon Test showed highly significant difference *** & ** respectively (Fig. 8).

## Discussion

In current study two citrus red mites, female specific genes (*PcVg* & *PcVgR*) were selected and their efficacies were observed by dsRNA leaf dip method. Results revealed that all the tested dsRNA of *PcVg* & *PcVgR* depicted a significant effect on female infertility. Furthermore, to assess the effect of target genes dsRNA combinations, the most operative concentration was selected for pest management by producing the infertile female. By using the combination (*PcVg* + *PcVgR*) with 1000 ng/ul concentration, we achieved up to 60.42% reduction in egg lying. Our findings help to develop a new gene specific and environment friendly approach, triggered by dsRNA to overcome the notorious pest *P. citri*.

Initially gene expression of target genes at different developmental stages was assessed. Target genes highly expressed in adult female whereas very low expression was noted in eggs. Eggs gene expression data is very close to previously reported research^18^ (Fig. 1).

Target genes dsRNA displayed high efficiency and showed very long lasting effect against dsRNA which ultimately caused the significant difference in egg laying as compared to control group (Figs 3A,B and 4A,B). The good efficiency of dsRNA can also be justified from our previous study^19^, when target genes dsRNA was applied before the sexually maturation, it caused the reduction in growth of sexually organ and abnormal development of ovary ultimately lead the insect toward female infertility. Our results can also be justified with previous reports which state that all the insects and oviparous species capability of reproduction totally rely on two major steps, 1) formation and deposition of *vitellogenin* (*Vg*) and 2) *vitellogenin receptor* (*VgR*), which is located on the surface of oocytes within clathrin-coated pits^20^. In insects, lipoprotein is traveled to its receptor site with the help of *VgR* and play a vital role in formation, development and functioning of oocytes^21^. Therefore, we conducted RNAi/dsRNA experiments to verify the gene function. Significant gene silence was noted, through the clear reduction in mRNA expression. It might inhibit the ovary function and development, which leads the insect toward female infertility. Same results have also been reported in silkworm^22^, cockroach^23^, tick^24^ fire ant^25^ and armyworm^26^. Cong *et al*., (2015) with his colleagues also reported that reduction in egg laying, delay in oviposition, failure in egg development and immature egg laying is due to knock down of *VgR* gene^27^. It is also documented that *Vg* gene is very important for the passage of fluorescently labeled cell wall pieces of *E. coli*, which play the imperative role in egg development in the ovaries^28^. *Vitellogenin* actively participates in trans-generational immune priming (TGIP), which can play dual functional protein like immunity and fecundity^29^. In addition, in honey bee *Vg* perform the different function like maintenance of workers and queen longevity and also manage the behavioral activity of by controlling the juvenile hormones^30–33^.

Post exposure effect of *PcVg* + *PcVgR* was noted on *P. citri* survival rate. Our findings revealed that life duration of treated mites was increased as compared to control group (ds-Egfp). It might be due to the considerable energy loss during reproduction, which ultimately causes reduction in life span^34^. Same kind of RNAi effect was noted on Lubber grasshopper life span in response to *Vg*-RNAi^35^. The effect of dsRNA (*PcVg* + *PcVgR*) reduces reproduction by declining mRNA level of *vitellogenin* and effects ovary development^36^. *Vg* mRNA knockdown, triggered by dsRNA also enhanced the life span of *Caenorhabditis elegans* ^37^.

We found non-significant difference in hatching rate as compared to control group but in case of dsRNA treated deutonymph, considerable reduction in egg laying and hatching was noted.

In molecular biology, RNAi is considered as advance, authentic and powerful tool with high specificity, efficiency, persistence and environment protection to overcome the insect pest population^38^. The use of RNAi to produce infertile female is unique approach and much safer than radiations and other pesticides. It is environment-friendly, keeps the females healthy, directly effects target gene and minimize the gene functioning such as reproduction ability. Our results showed that *PcVg*, *PcVgR* and their combined (*PcVg* + *PcVgR*) dsRNA, clearly reduced the egg laying percentage. Target genes we studied are also reported in other insect species that might open a new method for pest control.

During the research, we could not confirm the exact reason of female infertility and role of target genes and their pathways. Reasons for the reduction in egghatching in deutonymph and treated mites are also still unknown. In addition, numerous barriers exist for the commercial use of this technique, including the proper timing of dsRNA application, attainment of 100% infertility, fragment length and persistence and concentration. Moreover, until now RNAi effect of *Vg/VgR* dsRNA could not be maintained throughout the whole life of mites and still have a deficiency of field application by dsRNA transgenic plant or spraying of dsRNA reagent.

## Materials and Methods

### Rearing of Mites

Citrus red mites were collected from the sweet orange (Valencia) orchard, Huazhong Agricultural University, Hubei Wuhan, China. Orchard had never been sprayed with acaricide for the last ten years. Mites were kept in control condition (28 ± 1°, 80 ± 5% RH and 12:12 h L: D and considered to be a susceptible strain (SS). About 40 female mites were released on fresh leaves (3 mm diameter) and leaves were placed on 5-mm layer of sponge saturated with distilled water^7^.

### Selection of Target genes for RNAi

Two genes were selected (*PcVg* & *PcVgR*) based on the previous studies for RNAi studies^18^. Specific primers were designed by using the NCBI-Primer-BLAST **(** https://www.ncbi.nlm.nih.gov/tools/primer-blast/ **)** database (Table S1 & S2). A quantitative real-time PCR (qRT-PCR) analysis was performed to evaluate gene expression in different developmental stages and response to different concentrations of target gene dsRNA.

### Sequence verifications

Sequences of target genes, *PcVg* (GenBank Accession number: KC978893) and *PcVgR* (GenBank Accession number: KC978894) were selected. Total RNA was isolated from 80 female, adult mites. RNA samples were dissolved in 15 μL diethylpyrocarbonate (DEPC)-treated H2O and concentration was checked on NanoVue spectrophotometer. The quality of RNA was assessed by using the 1.5% agarose gel electrophoresis. Single strand cDNA was synthesized by using the commercially available kit (ThermoScientific, USA). PCR with target gene primers was run as; an initial denaturation was at 94 °C for 2 min, followed by 30 cycles of amplification at 94 °C for 30 s, 55–58 °C (depending on the annealing temperatures of the primers) for 30 s, 72 °C for 30 s and a final extension at 72 °C for 5 min. The PCR product was analyzed on gel electrophoresis and target bands were purified by Gel Extraction Kit (Omega, USA). Purified product was directly sent for sequencing (Invitrogen, shanghai, China).

### Double stranded RNA (dsRNA) preparation and feeding assays

Double stranded RNA (dsRNA) was synthesized from the open reading frame (ORF). Target genes fragment of 318 bp and 411 bp (*PcVg* & *PcVgR*) were selected. Control egfp fragment was characterized by using the egfp primers which contain the PUbnls EGFP vector^39^. Target genes dsRNA were synthesized by the commercially available kit (T7 RiboMAXTM Express RNAi System (Promega)). After preparation dsRNA product was purified by MEGA clear (Ambion) and stored at −80 °C until use. For RNAi, fresh leaves were washed with double distilled, cut into similar size and dried at at 55 °C for three minutes. Leaves were dipped into dsRNA solutions of different concentrations (250, 500, 750 & 1000 ng/ul) for 3 h. dsRNA infiltrated leaves were dried for 2–3 minutes in air laminar flow. Right after dsRNA-permeated leaves were put on water saturated sponge and 40 one day old female adult mites were released. dsRNA treated leaves with infested mites were incubated under control conditions and all biological experiments were repeated three times.

### Quantitative real-time PCR (qPCR) Analysis

Total RNA was isolated from 40 infested female mites collected every 48 hours. Single strand cDNA was synthesized by following the commercially available kit (ThermoScientific, USA) as mentioned above. Quantitative Real time PCR analysis was performed by using Universal SYBR Green iTaq™ Supermix (BioRad) on a Bio Rad iCycler according to company instructions. GAPDH was used as internal control gene^40^. Specific primers were designed ffor qPCR and reaction volume of 20 ul was used (0.8 ul of each primer, 2 ul cDNA, 6.4 ul ddH20 and 10 ul syber master mix) for quantification. Thermal cycler amplification conditions were maintained according to Xia *et al*.^41^. RT-qPCR data was analyzed according to Livak and Schmittgen^19^.

### Functional analysis

For analyzing the proper gene function 1000 ng/ul dsRNA concentration was chosen for both target genes and control group (Egfp). Leaves were dipped in dsRNA for 3 hours. Twenty pairs of one-day old mites were allowed to feed on treated leaves and freely mate. From treated leaves, infested mites are shifted to fresh leaves and old leaves were kept in the incubator for counting the number of eggs. After 7–8 days hatching rate was accessed. Leaves were changed after every 24 hours until 8 days.

### Synergistic effects of dsRNA on female fertility

Synergistic effect of 2 target genes (*PcVg* & *PcVgR*) was analyzed by mixing an equal volume of each dsRNA to achieve 1000 ng/ul concentration. Eggs laying and hatching were calculated by following the lab standardized method^38^.

### Determining technique factors for female infertility

To further confirm the combination of target genes (*PcVg* + *PcVgR*) dsRNA was used at the most effective concentration (1000 ng/ul) against different nymphal stages (deutonymph & protonymph). Additionally, the effect of dsRNA combination was also noted on the survival rate of adult females. In this regard, 200 newly adult female mites were selected and compared with the control group ds-Egfp.

### Data analyses

Data were statistically analyzed by analysis of variance (ANOVA) at P < 0.0001 according to Tukey’s test by using GraphPad prism 5.0 and represented as the mean ± SE. Survival rates were analyzed with log rank analysis and the Gehan–Breslow–Wilcoxon Test by using GraphPad Prism. An independent samples t-test was carried out for comparing the hatching rate of eggs per day, accumulative normalized egg hatching and proportion of the number of eggs laid per day.

## Electronic supplementary material

Supplementary Information
