# Supplementary material for: Biofunctional analysis of Vitellogenin and Vitellogenin receptor in citrus red mites, Panonychus citri by RNA interference
Source: Sci Rep. 2017 Nov 23;7:16123. doi: 10.1038/s41598-017-16331-3 (PMC5701056; doi:10.1038/s41598-017-16331-3)
Supplement: Supplementary file 1 — Supplementary Information [file 41598_2017_16331_MOESM1_ESM.pdf]

Biofunctional analysis of *Vitellogenin* and *Vitellogenin receptor* in citrus red mites, *Panonychus citri* by RNA interference

Muhammad Waqar Ali[1ǂ], Zhen-yu Zhang[1ǂ], Shuang Xia[1], Hongyu Zhang [1,*]

[1] Key Laboratory of Horticultural Plant Biology (MOE), State Key Laboratory of Agricultural Microbiology, Institute of Urban and Horticultural Entomology, College of Plant Science and Technology, Huazhong Agricultural University, Wuhan 430070, China. Correspondence and requests for materials should be addressed to H.Z. (Email: hongyu.zhang@mail.hzau.edu.cn

[ǂ] These two authors equally contributed in research work

Supplementary Tables

Table S1 Primers used to verify the expression of target genes in different developmental stages by qRT-PCR analysis

**Genes**	**Sequence**	**Gene expression in different body parts**
VG-F	AATGGTTGCGTTGAATACTGC	q-Real-time PCR
VG-R	CTCTGGGATGAGGGGAATGT	q-Real-time PCR
VgR -F	CTGTTTGGTATAAGCGTGCCA	q-Real-time PCR
VgR -R	CAACCACAGCCAATGCACAA	q-Real-time PCR
GADPH-F	CAACCAATTGTCTTGCTCCTTTG	q-Real-time PCR
GADPH-R	CGGTAGCGGCAGGTATAATG	q-Real-time PCR

Table S2 Primers used to amplify target gene fragments for dsRNA synthesis and qRT-PCR analysis

**Primers**	**Sequence**	**Purpose**
VG-F	CTTTCCTTGCTGGTAACTACAT	Gene cloning
VG-R	GATCTTTCGGATCTCCTCCT	Gene cloning
T7+*VG*-F	GGATCCTAATACGACTCACTATAGGCTTTCCTTGCTGGTAACTACAT	dsRNA synthesis
T7+*VG*-R	GGATCCTAATACGACTCACTATAGGGATCTTTCGGATCTCCTCCT	dsRNA synthesis
q-*VG*-F	ATCCCAGAGGAATCCGTTATC	q-Real-time PCR
q-*VG*-R	GTCTCGGGCTGAAAGGTGA	q-Real-time PCR
VgR-F	TCAGAGGGAAGTCAAATCCG	Gene cloning
VgR-R	TCCCCAATCAGACCAGAAC	Gene cloning
T7+*VgR* -F	GGATCCTAATACGACTCACTATAGGTCAGAGGGAAGTCAAATCCG	dsRNA synthesis

T7+*VgR* -R	GGATCCTAATACGACTCACTATAGGTCCCCAATCAGACCAGAAC	dsRNA synthesis
q-*VG* -F	CAAATGGAACCGCAAGAACA	q-Real-time PCR
q-*VG* -R	CATCTCCTGCCAGGTCAATCT	q-Real-time PCR
